# Breaking ground: nursing-led approach to alleviating constipation in Parkinson’s disease

**DOI:** 10.1186/s12877-023-04370-7

**Published:** 2023-10-13

**Authors:** Wenyao Geng, Mengdie Ren, Feng Zhao, Fuguo Yang, Heng Liu

**Affiliations:** 1 School of Nursing, Qingdao University, Qingdao, Shandong Province China; 2 Present Address: The Central Hospital of China Aerospace Corporation, Beijing, China

**Keywords:** Health Action Process Approach (HAPA), Nudge, Parkinson disease, Constipation, Delphi Technique

## Abstract

**Background:**

Constipation is one of the most common non-motor symptoms in PD patients, and the constipation, can appear before motor symptoms. Incorrect treatment of constipation in PD patients can result in colonic volvulus and pseudo intestinal obstruction, as well as a reduction in the therapeutic effect of anti-PD drugs due to absorption issues. There is, however, no comprehensive and scientific nursing intervention plan for PD patients’ constipation who are constipated.

**Methods:**

A multi-disciplinary nursing research group of five people was established to construct the first draft of intervention plan through literature review. We chose 15 experts from 7 universities and tertiary hospitals spread over 5 provinces (cities), including 4 neurologists, 9 clinical nursing specialists in neurology, 1 dietician, and 1 rehabilitator. Two rounds of consultations were held from April to July 2022 with 15 experts to screen and revise the indicators at each level, confirming their importance and feasibility at each level.

**Results:**

There were three primary indicators (pre-intentional stage, intentional stage, and action stage) in the two rounds of expert correspondence, nine secondary indicators (disease risk perception, adverse consequence expectation, self-efficacy and intention of action; action plan, coping plan and coping self-efficacy; produce healthy behaviors, maintain healthy behaviors, recover behaviors and recover self-efficacy), and 22 tertiary indicators.

**Conclusions:**

After the implementation of two rounds of Delphi method, the final formed constipation intervention program for PD patients provides the basis for clinical nursing practice, which has the characteristics of convenience, comprehensiveness, dependence, scientific and feasibility. Therefore, it has application and promotion value.

**Supplementary Information:**

The online version contains supplementary material available at 10.1186/s12877-023-04370-7.

## Background

Parkinson’s disease (PD) is a central nervous system (CNS) degenerative disease. There are currently 7 to 10 million people worldwide who have PD, and the prevalence of the disease is expected to rise by 50% by 2040 [1, 2]. China’s PD patient population is expected to reach 5 million by 2030, accounting for half of all PD patients worldwide [3]. PD is characterized by motor symptoms such as resting tremor, myotonia, bradykinesia, and postural balance disorders, while non-motor symptoms include hyposmia, constipation, sleep disturbances, depression, and anxiety [4]. Although PD is not fatal in and of itself, its complications and motor and non-motor symptoms can seriously impair a patient’s quality of life. In the early stages of PD, gastrointestinal dysfunction is the most prominent manifestation [5]. Constipation, on the other hand, is a common symptom of gastrointestinal dysfunction in PD, and it worsens as the disease progresses [6]. It has been reported that the prevalence of constipation in PD patients is 50%, with approximately 20% already experiencing constipation symptoms prior to diagnosis [4]. Constipation in PD has been described as persistent and difficult to treat [7]. First, patients with PD and their caregivers fail to pay attention to constipation symptoms, preferring simple medication over standardized and systematic treatment; second, a lack of standardized medication use and disease awareness leads to delayed treatment and wasted medical resources [8]. Inadequate management of constipation in PD patients not only reduces their quality of life, but can also result in PD malignant syndrome in severe cases [9]. As a result, constipation treatment and nursing interventions are an important part of managing non-motor symptoms in PD patients [10]. Although constipation is common in PD patients, there have been few studies on reasonable nursing intervention options for this patient’s constipation symptoms in clinical practice [11].

Health Action Process Approach (HAPA) is a stage-by-stage, continuous health behavior theory, proposed by German scholar Schwazer [12], that is a theoretical model useful for directing, forecasting, and explaining health behavior change. According to the theory, changes in health behavior are generally comprised of two processes. The motivational process (which primarily predicts the emergence of behavioral intentions) and the volitional process (which primarily emphasizes the prediction of behavioral change). Individual motivation and intention can be induced and generated risk perception, outcome expectation and self-efficacy, while self-efficacy for planning, coping and recovery has an impact on the volitional process; Schwazer also notes that health behavior change requires a continuous process of pre-intention, intention, and action stages. Foreign scholars have applied this theory to improve medicine adherence in rheumatoid patients, physical activity in college students, diabetes health promotion, and cancer screening, among other things [13–16]. According to the HAPA theoretical model, people at different stages of the process will face different problems and challenges, and will require different interventions to promote behavioral emergence or change, and that their action plans, coping plans, and coping self-efficacy will serve as mediators to bridge the gap between behavioral intentions and behavior production, resulting in long-term behavioral change [12]. However, it has been demonstrated that, despite having the intention and perceived ability to change behavior, i.e., to generate behavioral motivation, some individuals still struggle to initiate the action phase and make behavioral changes, resulting in a gap between expected health behaviors and actual behavior implementation.

The term of “nudge” was coined in 2008 by behavioral economists Thaler and Sunstein, who defined it as “predictable interventions that help people make more accurate and favorable choices without prohibiting any choices or significantly altering incentives in a way that is not readily apparent choices” [17]. A strategy for persuading people to change their behavior in the desired direction by using minor, more “implicit” actions [18]. Individuals use make two unique thinking processes to make judgements. The first is an unconscious, spontaneously formed system known as “intuitive thinking (System 1)”, and the second is a conscious, well thought-out system known as “rational thinking (System 2)” [19, 20]. Nudge works through influencing the unconscious and instinctive cognitive process, i.e., their “intuitive” thinking system (System 1), non-interference in individual choice, and promoting individual cognition to change, resulting in individual behavior change [18]. Some scholars classified helping strategies into nine categories [21], called “MINDSPACE strategy framework (Table 1)”, based on the mechanisms by which helping influences individuals’ cognitive and psychological activities, namely, Messenger, Incentives, Norms, Defaults, Salience, Priming, Affect, Commitments and Ego. According to Saghi, facilitation works by affecting an individual’s unconscious, automatic cognitive processes, i.e., by executing tiny tactics that do not significantly interfere with the individual’s freedom of choice. It takes only a small amount of effort on the part of the individual to promote behavioral motivation, intention, or cognitive changes that lead to behavioral changes [22]. However, most of the traditional intervention strategies based on health behavior theory affect the individuals’ conscious cognitive processes, that is the intervention affects the motivation, attitude, or knowledge level, etc. It has been recognized that certain behaviors are not conducive to health, but it is difficult for them to make behavioral changes [23]. In contrast, the nudge strategy (MINDSPACE) is to influence the unconscious cognitive process of an individual by using different situational forces (situational cues or situational stimuli), and then affect the individual’s motivation, attitudes, and intentions, so as to induce behavioral changes, make the individual consciously or unconsciously re-plan decision-making behaviors, and increase the possibility of the emergence or change of the individual health behaviors [17]. Therefore, when it comes to-promoting health behaviors, nudge strategies can be used as a supplement to HAPA theory (Health Behavior Theory) to better promote individual behavior change.

**Table 1:** Elements of the MINDSPACE Toolkit^a^

Elements	Description
Messenger	We are heavily influenced by who communicates information
Incentives	Our responses to incentives are shaped by predictable mental shortcuts such as strongly avoiding losses
Norms	We are strongly influenced by what others do
Defaults	We ‘‘go with the flow’’ of preset options
Salience	Our attention is drawn to what is novel and seems relevant to us
Priming	Our acts are often influenced by subconscious cues
Affect	Our emotional associations can powerfully shape our actions
Commitments	We seek to be consistent with our public promises, and reciprocate acts
Ego	We act in ways that make us feel better about ourselves

^**a**^ **Reproduced from Dolan et al.**

## Methods

### Establishing a research team

The research team included one nurse practitioner in charge of neurology, two associate chief physicians of neurology, one professor of postgraduate supervision, and one master of nursing student. The professor is in charge of subject idea control and quality control; other team members are in charge of searching for literature, organizing and analyzing data, and evaluating quality. This study follows the relevant EQUATOR guidelines (Additional file 1).

### Literature search and analysis

From January 2017 to May 2022, researchers searched the relevant literature in PubMed, Web of Science, and The Cochrane Library. The basic framework of the intervention program was determined by searching for a combination of subject terms and free words.

The following criteria were used to select the literature: one language (English); two study subjects with PD; and three study types (guidelines, expert consensus, evidence summaries, systematic reviews, and randomized controlled trials). Criteria for exclusion of literature: no full text was available; studies with poor literature evaluation. The quality of the included literature was assessed using the AGREE II evaluation tool for guidelines and the Australian JBI Centre for Evidence-Based Health Care’s literature quality evaluation tool for expert consensus, systematic reviews, and randomized controlled trials.

A two-person quality evaluation was used, with two graduate students conducting the quality evaluation, extracting and summarizing appropriate information on improving constipation in PD patients both nationally and internationally, and forming the first draft of the intervention protocol after discussion with the research team.

### Delphi expert correspondence

#### Development of the expert correspondence questionnaire

Following discussion in the research group, the expert correspondence questionnaire was developed based on the literature review and the purpose of the study. It is divided into four major sections: (1) Form instructions, which explain the background of this study as well as the form instructions and the deadline for returning the questionnaire. (2) Experts’ basic information. (3) Definition of relevant theories and concepts, as well as the introduction of the “Health Action Process Approach” and the “nudge” strategy. (4) Program consultation, including pre-intentional, intentional, and action phase intervention programs with 3 level one, 9 level two, and 22 level three entries.

### Selection of consulting experts

Purposive sampling was used to select specialists in neurology, gastrointestinal surgery, nutrition, rehabilitation medicine, and clinical nursing. Criteria for inclusion: (1) bachelor’s degree or higher, intermediate level or higher; (2) 10 years or more of relevant clinical experience; (3) understanding and knowledge of the Health Action Process Approach and nudge strategies; Four experts were highly motivated to participate and agreed to take part in several rounds of expert correspondence throughout the project.

This study included 15 experts from seven universities and tertiary hospitals in five provinces (cities), including Jilin, Liaoning, Jiangsu, Shandong, and Shanghai. There were 4 neurologists, 9 neurology clinical nursing specialists, 1 nutritionist, and 1 rehabilitator among them.

### Implementation of expert correspondence

This study had two rounds of expert consultation from April to July 2022, and the questionnaires were distributed in paper form or via email. Following the first round of expert consultation, the research team analyzed and revised the entries and contents in response to the experts’ comments or suggestions, and then created the second round of expert consultation questionnaire. Each round of questionnaires was distributed at 2–3 weeks intervals until the opinions of the experts converged. The importance and feasibility scores of 3.5 and below, as well as coefficients of variation greater than 0.25, were used to eliminate questionnaire entries, and the corresponding entries were revised after discussion by the research team, taking into account the experts’ opinions and suggestions.

### Statistical methods

SPSS 26.0 software was used for two-person data entry and analysis. Descriptive statistics were used to describe the means, standard deviations, expert positivity coefficients, expert authority coefficients, and the degree of expert opinion concentration of general information of experts. The expert positivity coefficient was expressed as the return rate of the questionnaire. Expert authority coefficient (*Cr*) = expert judgment coefficient (*Ca*) + expert familiarity (*Cs*)/2. The degree of expert opinion concentration was expressed as the mean of importance assignment. The degree of expert opinion coordination was expressed as coefficient of variation (*CV*) and Kendall’s coordination coefficient (Kendall’s *W*), and non-parametric tests were used to calculate the degree of expert opinion coordination, etc. Differences were considered statistically significant at *P* < 0.05.

## Results

### General information of experts

The final result of the two rounds of correspondence involved a total of 15 experts. The experts’ ages ranged from 32 to 63 (44.80 ± 9.57) years, with working years ranging from 10 to 45 (20.47 ± 11.38) years. There were six (40.00%) senior titles, seven (46.67%) associate titles, and two (13.33%) intermediate titles. There were nine PhDs (60.00%), three master’s degrees (20.00%), and three bachelor’s degrees (20.00%). Table 2 displays the basic information of experts.

**Table 2:** Basic expert information (n = 15)

Items	Number(%)
Age <40 40 ~ 50 >50	5(33.33) 5(33.33) 5(33.33)
Working years <15 15 ~ 30 >30	4(26.67) 8(53.33) 3(20.00)
Title middle level Associate Senior Positive Senior	2(13.33) 7(46.67) 6(40.00)
Qualifications Undergraduate Master Doctor	3(20.00) 3(20.00) 9(60.00)
Professional Direction Clinical nursing	9(60.00)
Clinical medicine Rehabilitation medicine Nutrition	4(26.67) 1(6.67) 1(6.67)

### Experts’ enthusiasm and authority degree

In the first round of expert consultation, 16 questionnaires were distributed and 15 valid questionnaires were returned, for a return rate of 93.8%; in the second round of expert consultation, 15 questionnaires were distributed and 15 valid questionnaires were returned, for a return rate of 100%. In this study, the Cr for the two rounds of consultation was 0.787.

### The degree of concentration and coordination of expert opinions

The mean of importance assignment of each item in the expert correspondence was 4.20 ~ 5.00 in round 1, and 4.40 ~ 4.87 in round 2; the coefficient of variation of importance rating of each item was 0 ~ 0.21 in round 1, and 0.07 ~ 0.17 in round 2; and the Kendall coordination coefficient of importance rating of each item was 0.313 in round 1, and 0.421 in round 2. Each item in the expert correspondence’s viability. The coefficient of variation for each item’s feasibility score was 0 ~ 0.19 in round 1, and 0 ~ 0.17 in round 2; the Kendall coordination coefficient for each item’s feasibility scores was 0.243 in round 1, and 0.254 in round 2. Table 3 shows the results.

**Table 3:** Kendall Harmony Coefficients and Test Results for Two Rounds of Expert Correspondence

Rounds	Importance Rating			Feasibility score		
	Kendall’s *W*	*χ* ^2^	*P*	Kendall’s *W*	*χ* ^2^	*P*
Round 1	0.313	144.82	<0.001	0.243	115.893	<0.001
Round 2	0.421	206.06	<0.001	0.254	124.349	<0.001

### Results of expert correspondence

Following the first round of expert correspondence, the study team revised the corresponding entries based on expert recommendations and opinions, which were then combined with clinical work. (See Table 4 for details). In the second round of correspondence, the experts made no objections, and the final draft of the intervention program for constipation in PD patients, which included 3 primary indicators, 9 secondary indicators, and 22 tertiary indicators, was completed, as shown in Table 5.

**Table 4:** The first round of Delphi experts’ letter of advice and modification

Content	Expert opinion	Modification
Delete content	Four experts suggested removing the intervention’s purpose because the tertiary indicators refer to specific interventions and do not need to describe each intervention’s purpose.	“Actively communicate with PD patients to understand their perception of constipation, and analyze the current situation with the patients to change the wrong perception.“
Merge or change the content	Five experts suggested that some of the tertiary indicators did not match the secondary indicators and that the order be merged or adjusted.	1. “1.3.1 Provide health education and inform patients of methods to intervene in constipation, such as adjusting dietary structure, increasing dietary fiber-rich foods, drinking more water, exercising appropriately, abdominal massage, and so on.“ should be reordered to the secondary index “1.2 Outcome expectation”; 2. “1.2.1 and 1.2.2” should be combined; should be changed to “Through successful cases, we can make patients realize the importance of active intervention on constipation; actively communicate with patients to relieve their psychological burden; and inform their families that PD can also lead to non-motor symptoms such as depression and anxiety, in order to help and supervise patients to find confidence in life.“
Modification content	1. Three experts proposed whether the three-level indicators should involve the intervention measures of patients’ family members. 2. Two experts suggested revising the details of items 2.1.1, 2.2.2 and 2.2.3, such as diet plan, exercise plan and defecation plan; Recommended therapeutic massage methods; Types, usage and precautions of therapeutic drugs	1. It has been revised to “ask family members for assistance when necessary” 2. (1)”2.1.1 Formulate and supervise diet, exercise, and defecation plans in collaboration with the patient’s condition and treatment plan, and in accordance with the patient’s wishes. Among them, the diet should ensure the intake of dietary fiber, energy, and water, and it is recommended to eat fruits and vegetables, with a water intake of at least 1.5-2.0L/day; encourage the patient to exercise regularly and appropriately, such as abdominal or pelvic floor muscle exercises; develop a regular defecation plan and develop good defecation habits, once a day, and the time of defecation should be within 2 hours after rising in the morning or after meals” (2) “2.2.2 Provide guidance and training to patients and their family members on how to perform abdominal massage and manipulation exercises; in collaboration with the actual patient, help to choose appropriate exercise methods to promote intestinal peristalsis, such as walking, Tai Chi, swimming, and so on; using Chinese medicine, perform acupuncture point external massage, abdominal acupuncture points (Zhonggui point, Shen Que point) manipulation?, and so on “; instruct patients to take warm water foot baths to stimulate intestinal peristalsis, and so on.“ (3) “2.2.3 When lifestyle changes do not improve constipation, patients should be promptly informed about the types of drugs and precautions for PD, as well as the use and precautions for constipation treatment drugs such as osmotic laxatives, stimulant laxatives, stool softeners, and so on.“

**Table 5:** A Parkinson’s disease-related constipation intervention program that combines the HAPA theory and nudge strategies

Index	nudge strategies	Importance score (‾x ± S)	CV	Feasibility score (‾x ± S)	CV
1.Pre-intentional stage		4.87 ± 0.35	0.072	5.0	0
1.1 Hazard perception		4.87 ± 0.35	0.072	4.87 ± 0.35	0.072
1.1.1 Take the initiative to communicate with PD patients in order to understand their perception of constipation and to assess the current situation with them	Priming	4.73 ± 0.46	0.097	4.60 ± 0.51	0.110
1.1.2 We explain the etiology, pathogenesis, clinical manifestations, and health risks of PD constipation to patients via phone, video, and face-to-face communication, and emphasize the importance of early intervention	Messenger Priming	4.53 ± 0.52	0.114	4.67 ± 0.49	0.105
1.1.3 We explain to patients the etiology, pathogenesis, clinical manifestations, and health risks of PD constipation via phone, video, and face-to-face communication, and emphasize the importance of early intervention	Messenger Priming	4.47 ± 0.64	0.143	4.40 ± 0.63	0.144
1.2 Results expectations		4.60 ± 0.51	0.110	4.60 ± 0.51	0.110
1.2.1 Make patients aware of the importance of active intervention for constipation through successful cases; communicate positively with patients to relieve their psychological burden; and inform family members that PD can also cause non-motor symptoms such as depression and anxiety in order to assist and supervise patients in regaining confidence in their lives	Norms Affect	4.87 ± 0.35	0.072	4.73 ± 0.46	0.097
1.2.2 Inform patients about ways to treat constipation through health education, such as changing their lifestyle, adjusting their diet, increasing foods high in dietary fiber, drinking more water, exercising appropriately, and abdominal massage, among other things	Messenger Priming	4.60 ± 0.63	0.137	4.60 ± 0.51	0.110
1.3 Self-efficacy and intention for action		4.67 ± 0.49	0.105	4.73 ± 0.46	0.097
1.3.1 Constipation information brochures are distributed on a regular basis, as well as instructional videos on abdominal twisting exercises and information brochures for patients’ families	Messenger Priming	4.60 ± 0.63	0.137	4.60 ± 0.51	0.110
2. Intentional stage		4.87 ± 0.35	0.072	4.87 ± 0.35	0.072
2.1 Action planning		4.80 ± 0.41	0.086	4.87 ± 0.35	0.072
2.1.1 Formulate and supervise diet, exercise, and defecation plans in collaboration with the patient’s condition and treatment plan, and in accordance with the patient’s wishes. Among them, the diet should ensure the intake of dietary fiber, energy, and water, and it is recommended to eat fruits and vegetables, with a water intake of at least 1.5-2.0 L/day; encourage the patient to exercise regularly and appropriately, such as abdominal or pelvic floor muscle exercises; develop a regular defecation plan and develop good defecation habits, once a day, and the time of defecation should be within 2 h after rising in the morning or after meals	Messenger Salience	4.80 ± 0.41	0.086	4.67 ± 0.49	0.105
2.2 Coping planning		4.73 ± 0.46	0.097	4.73 ± 0.46	0.097
2.2.1 Discuss potential barriers to program implementation with patients and families, and work with them to develop a plan to address them	Ego Salience	4.73 ± 0.46	0.097	4.60 ± 0.51	0.110
2.2.2 Provide guidance and training to patients and their family members to perform abdominal massage and manipulation exercises; combined with the actual patient, according to the existing guidelines and expert consensus on PD exercise prescription, help to choose appropriate exercise methods to promote intestinal peristalsis, such as walking, playing Tai Chi, swimming, etc.; using Chinese medicine, perform acupuncture point external massage, abdominal acupuncture points (Zhong-Gui point, Shen-Que point) manipulation, etc.; instruct patients to take warm water foot baths to promote intestinal peristalsis, etc.	Messenger Salience Incentives	4.80 ± 0.56	0.117	4.67 ± 0.62	0.132
2.2.3 When lifestyle changes do not improve constipation, patients should be promptly informed about the types of drugs and precautions for PD, as well as the use and precautions for constipation treatment drugs such as osmotic laxatives, stimulant laxatives, stool softeners, and so on	Messenger Salience	4.40 ± 0.74	0.167	4.40 ± 0.74	0.167
2.3 Coping self-efficacy		4.47 ± 0.74	0.166	4.60 ± 0.63	0.137
2.3.1 Maintain contact with the patient and offer assistance based on the patient’s specific situation: reduce symptoms in constipation patients and improve prevention in non-constipation patients	Incentives Ego	4.47 ± 0.74	0.166	4.40 ± 0.74	0.167
2.3.2 Encourage successful constipation intervention patients to share their experiences and assist one another via channels such as online WeChat groups, WeChat public numbers, brochures, and offline patient exchange meetings	Norms	4.53 ± 0.52	0.114	4.67 ± 0.49	0.105
2.3.3 Encourage the patient to commit to following the diet and bowel program; inform the patient that having a bowel movement once every two to three days is normal as long as it is soft and easy to pass	Incentives Commitments	4.64 ± 0.50	0.107	4.53 ± 0.64	0.141
3. Action stage		4.87 ± 0.35	0.072	4.93 ± 0.26	0.052
3.1 Generative behavior		4.67 ± 0.49	0.105	4.73 ± 0.46	0.097
3.1.1 Instruct the patient to keep a diet and bowel movement diary (with family assistance if needed)	Ego	4.80 ± 0.56	0.117	4.80 ± 0.41	0.086
3.1.2 Encourage patients to share their daily diet and bowel diary via WeChat groups, pinning groups, face-to-face communication, and other means	Ego Norms	4.60 ± 0.63	0.137	4.60 ± 0.63	0.137
3.1.3 For those who are constipated and have not had a bowel movement in more than 5 days, the doctor may prescribe a corkage or glycerin enema, as well as a small amount of reserved enema if necessary (with the assistance of family members if necessary)	Messenger	4.60 ± 0.74	0.160	4.60 ± 0.74	0.160
3.2 Maintain behavior		4.87 ± 0.35	0.072	4.67 ± 0.62	0.132
3.2.1 Display warning signs in prominent places to remind patients to follow their diet, exercise, and bowel movement plans	Salience	4.60 ± 0.63	0.137	4.47 ± 0.74	0.166
3.2.2 Encourage patients to keep a bowel and diet diary on a regular basis (ask family members to assist if necessary)	Incentives	4.87 ± 0.35	0.072	4.73 ± 0.46	0.097
3.2.3 Establish a follow-up register, make regular follow-up visits, assess patients’ intervention effects and problems, and deal with them in a timely manner with specific situations using WeChat groups, phone calls, and face-to-face communication,	Affect Ego	4.60 ± 0.51	0.116	4.53 ± 0.52	0.114
3.3 Recovery behavior and recovery self-efficacy		4.47 ± 0.52	0.110	4.60 ± 0.51	0.110
3.3.1 Encourage patients to self-motivate by telling themselves their bowel movement plan for the day every day through self-talk and, upon completion, saying to themselves, “I stuck to the plan, I’m great,“ and so on	Incentives Ego	4.60 ± 0.51	0.110	4.53 ± 0.52	0.114
3.3.2 To validate the patients’ results and boost their self-esteem; to determine the reasons for and provide countermeasures for those patients who do not persist	Incentives Ego	4.60 ± 0.51	0.110	4.73 ± 0.46	0.097
3.3.3 Communicate with patients on a regular basis to understand their thoughts and feelings, and provide emotional guidance and support as needed. Encourage patients to engage in social activities and recognize their own worth. Communicate with family members on a regular basis to encourage them to strengthen the company of patients and to foster a peaceful family environment in order to reduce patients’ loneliness	Affect	4.87 ± 0.35	0.072	4.73 ± 0.46	0.097

### Ethical review

Ethical approval was granted by the relevant university and university human research ethics committees.

## Discussion

### A constipation intervention program for PD patients based on a combination of the health action process approach and nudge strategies is scientifically sound

Through a literature review and two rounds of expert correspondence, the theoretical basis and basic framework of a constipation intervention program for PD patients combining the Health Action Process Approach and nudge strategies were identified in this study, and the significance and feasibility of the intervention program were determined. The correspondence work demonstrated that the experts had rich clinical practice experience as well as authority and representativeness by 15 experts from clinical and university settings, all with rich working experience, 13 (86.67%) with associate senior titles and above, 12 (80.00%) with master’s degrees and above, and 12 (80.00%) with ≥ 15 years of working experience in neurology-related fields. The expert correspondence results revealed that the experts’ authority coefficient Cr in both rounds was greater than 0.7, indicating that the experts had a high-level authority and were scientific. In the two rounds, the recovery rate of expert questionnaires was 93.8% and 100%, respectively, and the rate of expert opinions raised was 80.00% and 20.00%. This demonstrates that the experts are highly motivated. The Kendall’s *W* for the importance rating of the two rounds of expert correspondence was 0.313 and 0.421, respectively, with coefficients of variation of 0.25 for each item, indicating that the experts’ opinions tend to be consistent and the correspondence results are more scientific. The intervention protocol entries constructed in this study were not based on personal opinion, but were consistent with clinical data from patients with PD.

### An intervention program for constipation symptom management in PD patients combining HAPA theory and nudge strategies is easy to implement and useful

With the continuous development of society and economy, the gradual improvement of people’s living standards, PD has become a problem that cannot be ignored. At present, clinical treatment research on PD has been carried out, but the health management of most patients adopts health education in healthy way. Although it will improve patients’ willingness to control their health behaviors, it ignores whether patients’ health behaviors are really changed. Due to the long duration of PD, patients cannot form long-term self-management of health behaviors through a single health education [24, 25]. On this basis, this study combined health behavior process-oriented theory and nudge strategies to construct a nursing intervention program suitable for constipation in PD patients, which enhanced the PD utilization stage sector model of intervention in constipation patients, and increase the possibility of the patient’s health behavior to generate or change through subtle nudge strategies (MINDSPACE strategy framework). By promoting the nudge strategy, patients are prompted to start the motivation process, generate belief and learning motivation improve self-control, fully explore the potential of independent learning, increase the motivation of patients to learn disease-related knowledge, further prompt the belief and motivation of patients to control diet behavior, and then improve the motivation of patients to change behavior and initiate the will process of behavioral change, and promote the emergence of healthy behaviors.

1. **An intentional pre-stage intervention program for patients with constipation in PD**. The intervention plan in the pre-intention stage is mainly through simple information reminder. For patients with PD, imitating and prominent promotion strategies are mainly adopted to activate the motivation process of patients by influencing the unconscious cognitive process of patients and promoting the formation of constipation symptom management intention. **① Counter risk perception**: Initiate behavioral beliefs about constipation symptom management by providing information support to patients and weighing the pros and cons. The emergence and change of individual health behaviors can be influenced by subconscious cues such as speech, and individual health goals can be initiated through text judgment tasks [26]. Thus, the intervention protocols developed in this study shaped patients’ perceptions of PD and promoted their intention to produce healthy behavior changes through a number of videos or articles, warning charts, and other educational tools. **② Expectations for outcomes**: Enhance behavioral intent for constipation symptom management by providing significant information, standardizing reference groups, and guiding positive beliefs. Research has shown that prominent information, such as “traffic light” labels, can effectively promote choices about individual health behaviors [21]. Red and graphic overlays have a more consistent effect on the emergence and maintenance of healthy behaviors compared to more traditional black numbers [27]. Therefore, the intervention protocol developed in this study was designed to educate patients through the use of more directional and targeted information, such as the homemade “Constipation Treatment Information Booklet”. **③ Self-efficacy and behavioral intent**: Improve self-efficacy by providing emotional support to patients. Therefore, in the intervention protocol developed in this study, patients are encouraged to express their feelings, clarify their problems, listen to their anxiety, and strive to reduce anxiety. Their family members or other significant family members are asked to raise expectations, resulting in positive expectations of outcomes. Family members are encouraged to participate in the management of constipation symptoms, increase the number of patients accompanying them, and provide emotional relief when necessary, so as to improve patients’ sense of self-efficacy and lead to constipation symptom management behavior [28]. This will improve patients’ sense of self-efficacy and motivate them to develop constipation symptom management behaviors.
2. **An intervention plan for the intention stage of constipation symptom management in PD patients**. The intentionality phase of the intervention program focuses on reinforcing or consolidating the intentionality of constipation symptom management in patients with PD, primarily using significance and normative references in promoting strategies. Unlike intervention plans in the pre-intentional phase, the use of saliency strategies in the intentional phase is mainly reflected in the intervention of PD patients through simplified information. **①Targeted action plan**: By giving patients significant information support and public commitment, enhance patients’ perceptual behavioral control and promote the transformation of behavioral intentions into behaviors. The results of an online survey in the United Kingdom showed that poster campaigns using more images and less text were more helpful in promoting hand hygiene-related behaviors [29]. Therefore, the intervention program developed in this study promotes behavior generation through a one-to-one approach to simplified milestones and personalized meal plans, fully enhancing the external support process of patient behavior by will and increasing patient receptiveness to information by overcoming the gap between intention and action. At this stage, we also encourage patients to make a public commitment to adhere to defecation and scientific diet, strengthen patients’ self-discipline determination, and motivate healthy behavior change, thus contributing to the realization of healthy behavior in the management of constipation symptoms. ② **For coping planning**: By giving patients significant information support, regulating reference behaviors, enhancing confidence, and facilitating the transformation of behavioral intentions into actions. Individual decision making is also influenced by others’ behavior, i.e., normative reference [30]. For example, by vigorously carrying out various sports organization activities, people’s willingness to engage in sports activities is enhanced and people’s exercise behavior is further developed. Therefore, in the intervention plan formulated in this study, patients with good constipation symptom management behavior were invited to share the difficulties encountered in the process of we-chat group and the coping methods to provide examples; Evaluate with the patient the implementation of the diet plan and target last week, evaluate with the patient the implementation of the intestinal plan and other target plans last week, summarize and analyze the obstacles; At the same time, according to the completion of the patient’s diet plan and goals, the researcher will summarize and re-formulate the completion of the diet plan and goals in the next stage with the patient. At the same time, the researchers summarized and reformulated the next stage of the patients based on the completion of the patients’ diet plan and goals. **③ Coping self-efficacy**: Improve patients’ coping self-efficacy by providing them with adequate emotional support. The intervention plan developed in this study, by maintaining contact with patients, providing help according to their specific situation, listening to their psychological feelings in managing constipation symptoms, encouraging them to express their bad emotions, and informing them, healthcare professionals provide guidance throughout the process to ensure team generation and maintenance behavior; Researchers and family members become important supporters of patients, timely detect patients’ bad emotions, guide patients to learn ways and skills of communication and expression, talk to family and friends, and give patients positive and positive feedback. Researchers and patients’ families become important advocates for patients and guide patients in learning communication and presentation skills that enable them to talk to family and friends and give them positive emotional support.

(3) **An intervention plan for the stage of constipation symptom management in PD patients**. The action phase of the intervention program focuses on motivating people with PD to produce, maintain, or restore healthy behaviors to manage constipation symptoms, primarily using self-image and motivation derived from push strategies. **① Behavior generation**: Promote the transformation of behavioral intention into behavior by providing significant information support. Therefore, the intervention protocol developed in this study enabled patients to adhere to the daily recording and evaluation of dietary control punched in a diet and bowel diary, with information regularly sent for constipation symptom management, and guided patients to do constipation symptom management warning cards, food wall maps, and nutrition labels, adhere to patients’ bowel studies and daily recording and evaluation of diet or activity. **② Guiding patients to perceive the benefits of their own adverse health behaviors**: this will enhance their attitude to respond positively to behavior-changing situations, and drive their confidence and social support. Health behavior of constipation symptom management in patients with Parkinson’s disease. **③ Rehabilitative behavior and self-efficacy**: Enhance the self-efficacy of rehabilitative behavior by giving patients prominent information and emotional support. In the intervention plan formulated in this study, the researchers reviewed and analyzed the achievement of the patients’ goals, and affirmed and encouraged the patients. Family members actively identify patients’ bad emotions in the process of behavior change, and provide comfort and relief in time, reducing the possibility of behavior interruption caused by bad emotions. Family members are also encouraged to express their commitment to patients with constipation symptom management as well as diet and exercise management. At the same time, family members are encouraged to do the following work, and family members are encouraged to supervise the patient’s daily constipation management and cook for the patient when necessary. During this period, researchers need to strengthen communication with patients, enhance the sense of trust, and provide appropriate guidance and emotional support for patients’ difficulties in order to improve the self-efficacy of patients’ rehabilitation. Constipation symptom management is a long-term and continuous process. Members of the research team will understand the status quo of constipation symptom management of patients through regular follow-up visits, answer patients’ questions in a timely manner, encourage patients to make correct behavioral changes in constipation symptom management, enhance patients’ confidence in adhering to their own behaviors, and prevent the interruption of constipation symptom management behaviors. For patients who may experience behavioral disruption, self-image perception guidance helps them recall the benefits of constipation symptom management, acknowledge and praise their efforts and achievements, improve patients’ awareness of constipation symptom management, enhance their confidence in constipation symptom management, and thus promote the emergence or maintenance of behaviors [31]. Informational support and emotional strategies were used in all three phases of the project to intervene with people with PD. An individual’s emotional response to environmental cues such as words, images, and events has been shown to alter health behaviors. Therefore, this study promotes the emergence and maintenance of healthy behaviors for constipation symptom management by encouraging family members to work with patients to manage constipation symptoms, providing health education to patients and their families, improving patients’ social support, and establishing or restoring patients’ self-efficacy through positive and positive emotional support.

The constipation intervention program in based on the Health Action Process Approach and nudge strategies that emphasizes assessment, guidance, and support for patients and are practical, it promotes patients to move from passive to active behavior and improve their self-efficacy. Through small, minor strategies based on the formation of patients’ health behavior intentions. Therefore, through literature review and expert correspondence, our term developed this protocol in combination with the characteristics of non-motor symptoms (gastrointestinal dysfunction) of PD and the needs of patients, in order to provide some reference for improving constipation symptoms of PD patients and actual clinical care.

### Limitations

(1) This study did not verify the long-term effects of the application of this intervention regimen. To more accurately observe the difference in effect between the intervention group and the control group.

(2) Recommendations: Future studies need to further improve the study design, expand the number of samples and sample sources, and further validate the effects and results if conditions permit, in order to promote the generalization of the research findings.

(3) In the future, randomized controlled trials will be needed to confirm the feasibility and effectiveness of this protocol.

## Conclusions

Through a literature review and two rounds of expert consultation, we developed a constipation intervention program for PD patients in our study. The protocol includes 3 primary indicators, 9 secondary indicators, and 22 tertiary indicators that are scientifically rigorous and serve as a foundation for clinical improvement of constipation-related care measures in PD patients. Patients with PD constipation are seriously affected physically and mentally. It has been reported that reasonable and effective nursing intervention can improve the constipation of patients with PD and improve their quality of life. The nursing intervention constructed in this study is a new nursing model, mainly by awakening the unconscious cognitive process of patients, and making nursing plans according to the actual situation. Through individualized diet, exercise and medication care, help patients develop good living habits and improve constipation. In terms of diet, patients should eat more high-fiber foods and fresh fruits and vegetables to improve constipation. Proper exercise can effectively promote gastrointestinal peristalsis and improve constipation. At the same time, appropriate exercise can also enhance the immunity and resistance of patients, which is conducive to the rehabilitation of patients. Medication guidance can ensure the effectiveness of clinical medication and improve patient’s medication compliance. Psychological nursing can eliminate patients’ negative emotions, make patients face treatment positively, and help to establish a harmonious doctor-patient relationship, so as to improve patients’ satisfaction with nursing.

In summary, the nursing model constructed in this study has an ideal application effect, and constipation in elderly patients with PD has been significantly improved, which is worthy of clinical application and promotion.

### Electronic supplementary material

Below is the link to the electronic supplementary material.

Supplementary Material 1

## Data Availability

The datasets used during the current study are available from the corresponding author on reasonable request.
