# Supplementary material for: Breaking ground: nursing-led approach to alleviating constipation in Parkinson’s disease
Source: BMC Geriatr. 2023 Oct 13;23:657. doi: 10.1186/s12877-023-04370-7 (PMC10571412; doi:10.1186/s12877-023-04370-7)
Supplement: Supplementary file 1 — Supplementary Material 1 [file 12877_2023_4370_MOESM1_ESM.pdf]

1 The RIGHT Checklist

Section/ topic	No.	Item	Assessment	Page(s)	Note
Basic information					
Title/ subtitle	1a	Identify the report as a guideline, that is, with "guideline(s)" or "recommendation(s)" in the title.	NO		
	1b	Describe the year of publication of the guideline.	NO		
	1c	Describe the focus of the guideline, such as screening, diagnosis, treatment, management, prevention or others.	YES	1	
Executive summary	2	Provide a summary of the recommendations contained in the guideline.	NO		
Abbreviations and acronyms	3	Define new or key terms, and provide a list of abbreviations and acronyms if applicable.	YES	1	
Corresponding developer	4	Identify at least one corresponding developer or author who can be contacted about the guideline.	YES	Title page	
Background					
Brief description of the health problem(s)	5	Describe the basic epidemiology of the problem, such as the prevalence/ incidence, morbidity, mortality, and burden (including financial) resulting from the problem.	YES	1	
Aim(s) of the guideline and specific objectives	6	Describe the aim(s) of the guideline and specific objectives, such as improvements in health indicators (e.g., mortality and disease prevalence), quality of life, or cost savings.	YES	1	
Target population(s)	7a	Describe the primary population(s) that is addressed by the recommendation(s) in the guideline.	YES	1	
	7b	Describe any subgroups that are given special consideration in the guideline.	NO		
End- users and settings	8a	Describe the intended primary users of the guideline (such as primary care providers, clinical specialists, public health practitioners, program managers, and policy-makers) and other potential users of the guideline.	YES	2-3	
	8b	Describe the setting(s) for which the guideline is intended, such as primary care, low- and middle-income countries, or in-patient facilities.	YES	2-3	
Guideline development group	9a	Describe how all contributors to the guideline development were selected and their roles and responsibilities (e.g., steering group, guideline panel, external reviewer, systematic review team, and methodologists).	YES	Title page	
	9b	List all individuals involved in developing the guideline, including their title, role(s) and institutional affiliation(s).	YES	Supplementary material	
Evidence					
Healthcare questions	10a	State the key questions that were the basis for the recommendations in PICO (population, intervention, comparator, and outcome) or other format as appropriate.	YES	1	
	10b	Indicate how the outcomes were selected and sorted.	YES	2-3	

Systematic reviews	11a	Indicate whether the guideline is based on new systematic reviews done specifically for this guideline or whether existing systematic reviews were used.	YES	2	
	11b	If the guideline developers used existing systematic reviews, reference these and describe how those reviews were identified and assessed (provide the search strategies and the selection criteria, and describe how the risk of bias was evaluated) and whether they were updated.	YES	2-3	
Assessment of the certainty of the body of evidence	12	Describe the approach used to assess the certainty of the body of evidence.	YES	2-3	
Recommendations					
Recommendations	13a	Provide clear, precise, and actionable recommendations.	YES	Supplementary material	
	13b	Present separate recommendations for important subgroups if the evidence suggests that there are important differences in factors influencing recommendations, particularly the balance of benefits and harms across subgroups.	NO		
	13c	Indicate the strength of recommendations and the certainty of the supporting evidence.	NO		
Rationale/ explanation for recommendations	14a	Describe whether values and preferences of the target population(s) were considered in the formulation of each recommendation. If yes, describe the approaches and methods used to elicit or identify these values and preferences. If values and preferences were not considered, provide an explanation.	YES	3-4	
	14b	Describe whether cost and resource implications were considered in the formulation of recommendations. If yes, describe the specific approaches and methods used (such as cost-effectiveness analysis) and summarize the results. If resource issues were not considered, provide an explanation.	YES	3-4	
	14c	Describe other factors taken into consideration when formulating the recommendations, such as equity, feasibility and acceptability.	YES	3-4	
Evidence to decision processes	15	Describe the processes and approaches used by the guideline development group to make decisions, particularly the formulation of recommendations (such as how consensus was defined and achieved and whether voting was used).	YES	2-3	
Review and quality assurance					
External review	16	Indicate whether the draft guideline underwent independent review and, if so, how this was executed and the comments considered and addressed.	NO		

Quality assurance	17	Indicate whether the guideline was subjected to a quality assurance process. If yes, describe the process.	YES	2	
Funding, declaration and management of interest					
Funding source(s) and role(s) of the funder	18a	Describe the specific sources of funding for all stages of guideline development.	YES	Title page	
	18b	Describe the role of funder(s) in the different stages of guideline development and in the dissemination and implementation of the recommendations.	NO		
Declaration and management of interest	19a	Describe what types of conflicts (financial and non-financial) were relevant to guideline development.	YES	Title page	
	19b	Describe how conflicts of interest were evaluated and managed and how users of the guideline can access the declarations.	NO		
Other information					
Access	20	Describe where the guideline, its appendices, and other related documents can be accessed.	YES	2	
Suggestions for further research	21	Describe the gaps in the evidence and/or provide suggestions for future research.	YES	3-4	
Limitations of the guideline	22	Describe any limitations in the guideline development process (such as the development groups were not multidisciplinary or patients' values and preferences were not sought), and indicate how these limitations might have affected the validity of the recommendations.	YES	3-4	
